# Revolutionizing Huntington’s Disease Treatment: Breakthroughs in AAV-Mediated Gene Therapy

**DOI:** 10.3390/cells14191514

**Published:** 2025-09-28

**Authors:** Pedram Moeini Gavgani, Mario García-Domínguez

**Affiliations:** 1 DNA & RNA Medicine Division, CIMA-Universidad de Navarra, 31008 Pamplona, Spain; 2 Program of Immunology and Immunotherapy, CIMA-Universidad de Navarra, 31008 Pamplona, Spain; 3 Department of Immunology and Immunotherapy, Clínica Universidad de Navarra, 31008 Pamplona, Spain; 4 Centro de Investigación Biomédica en Red de Cáncer (CIBERONC), 28029 Madrid, Spain

**Keywords:** adeno-associated viral vector, Huntington’s disease, huntingtin gene, gene therapy, neurodegenerative disorders

## Abstract

Huntington’s Disease (HD) is an inherited neurodegenerative condition caused by an expansion of CAG repeats in the Huntingtin (HTT) gene, leading to a toxic form of the HTT protein. Despite advances in understanding the disease and developing symptomatic treatments, effective therapies for modifying its progression remain limited. Among emerging and novel treatments for central nervous system (CNS) disorders, gene therapy (GT), particularly using adeno-associated virus (AAV)-mediated gene delivery, holds great promise. Numerous preclinical and clinical trials are exploring the benefits of AAVs for treating neurodegenerative and genetic diseases. However, while widely used and investigated in rare and genetic disease treatment, AAVs’ potential for HD treatment remains underexplored. The absence of a comprehensive collection of previous reports, advancements, and methodologies regarding exclusively AAV-mediated GT for HD is notable and prompted us to address this gap. The current review compiles the available and emerging information regarding the application of AAVs in HD therapy, outlines the promise of this approach, and highlights the necessity of conducting further studies to achieve efficient HD treatment. The authors hope that the current review will guide further research to unlock the full potential of AAVs in treating HD.

## 1. Introduction to Huntington’s Disease

Huntington’s disease (HD) is an autosomal dominant neurodegenerative pathology with complete penetrance, caused by a CAG trinucleotide repeat expansion in the Huntingtin (*HTT*) gene, located on chromosome 4 [1]. This genetic alteration leads to the production of a mutated form of the HTT protein (known as mHTT), which is neurotoxic [1].

In 1842, Charles Oscar Waters reported the first case of Huntington’s chorea (HC) which was later termed as HD after George Huntington provided a powerful description in 1872 [2]. Over the years, our understanding of this disease has significantly improved, especially after the identification of the *HTT* gene and the CAG repeat mutation in 1993 [3]. These discoveries have been crucial for the development of diagnostic tools and have driven ongoing research into effective therapies [4].

HTT is a widely expressed, multifunctional protein that is essential for maintaining cellular homeostasis, comprising vesicular trafficking, endocytosis, axonal transport, transcriptional regulation, and autophagy [5,6]. HTT also associates with other protein complexes, enabling intracellular transport processes and sustaining synaptic activity [7]. In addition, HTT controls the transcription of neuronal survival-related genes by interacting with some transcription factors and chromatin-modifying proteins [8].

In HD patients, the mHTT protein is present at abnormally elevated levels [9], with the associated mechanisms outlined in Section 1.1. mHTT disrupts normal interactions between proteins, which causes misfolded protein aggregates to build up in both nuclear and cytoplasmatic compartments [10]. These aggregates destabilize the ubiquitin-proteasome system and autophagic pathways, impairing the clearance of damaged proteins and organelles [11]. Additionally, by interfering with several transcription factors (e.g., CBP) and disrupting histone acetylation, mHTT disrupts transcriptional regulation, causing broad alterations in neuronal transcriptional profiles [12,13]. mHT progressively impairs mitochondrial bioenergetics and perturbs Ca^2+^ signaling, thereby amplifying oxidative stress and excitotoxicity and promoting excitotoxic neuronal death [14]. Finally, mHTT strongly impacts synaptic integrity and function. Abnormal protein interactions prevent the proper trafficking and recycling of synaptic vesicles, regulate inhibitory signaling, causing altered release of neurotransmitter, changes in postsynaptic receptor composition, and impairments in synaptic plasticity [15,16,17].

### 1.1. Genetics and Epigenetics

**Figure 1:**
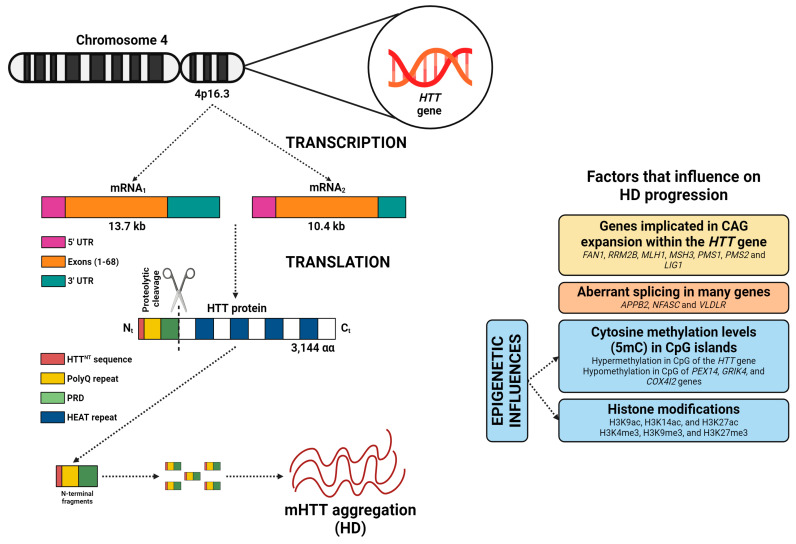
The *HTT* gene contains 67 exons and produces two mRNAs (10.4 and 13.7 kb), differing by a 3′ UTR sequence. The resulting HTT protein includes an N-terminal region with a nuclear export signal (HTTNT), a polyglutamine repeat (polyQ), and a proline-rich domain (PRD), followed by clusters of anti-parallel alpha-helical HEAT repeats. In HD, the proteolytic cleavage of N-terminal fragments by peptidases is more frequent, and these fragments are believed to play a role in disease pathogenesis (by formation of mHTT protein). However, numerous factors significantly influence disease progression, including the involvement of genes associated with DNA repair, aberrant splicing, methylation levels of cytosines (5mC) in CpG islands across some genes, and the histone methylation and acetylation levels. Image adapted from BioRender (https://www.biorender.com/).

The *HTT* gene, located on chromosome 4 (4p16.3), encompasses 67 exons and extends over a physical distance of 180 kb of genomic DNA (Figure 1), with transcription occurring from the telomere to the centromere [18]. Differential polyadenylation of the 3′ untranslated region (3’UTR), encoded in the final exon, results in the production of two transcripts (10.4 kb and 13.7 kb), which are found in some cell types, including neurons [19]. HD is caused by a dominant mutation with complete penetrance, leading to an increase in CAG trinucleotide repeats in the *HTT* gene, specifically within the first exon (Figure 1). This mutation results in the biosynthesis of an HTT protein with an abnormally elongated polyQ sequence [20]. The wild-type HTT protein (23 CAG repeats) is composed of 3144 amino acids (348 kDa), and is conserved across mammalian species, indicating its role in survival [21]. The HTT protein comprises an N-terminal region that includes various elements [22]: (i) the HTTNT, a 17 amino acid sequence, fundamental for nuclear export and endoplasmic reticulum targeting; (ii) the polyQ repeat, linked to pathology initiation; (iii) a proline-rich domain (PRD) that evokes protein aggregation. Proteolytic cleavage of N-terminal fragments (HTTNT, polyQ, and PRD) is more frequent in HD, and these fragments play a crucial role in HD pathogenesis [23]. The rest of the HTT protein is structured into clusters of anti-parallel alpha-helical HEAT repeats, which serve as scaffold motifs for other protein macromolecules [22]. The mHTT protein, primarily its N-terminal fragments, can form aggregates and interfere with cell signaling, axonal transport, transcription, translation, and synaptic function, while promoting apoptosis. These anomalies contribute to neuronal dysfunction and the progression of HD [24,25].

In unaffected individuals, CAG repeat lengths usually range from 17 to 20 [26]. However, once the CAG repeats reach 27 or more, the risk of developing HD increases exponentially [27,28,29,30]. Moreover, Handsaker et al. (2025) [31], using single-cell HTT-CAG and RNA sequencing, demonstrated that striatal projection neurons undergo somatic HTT CAG expansion from 40–45 to 100–500+ repeats. Expansions up to ~150 CAGs were largely tolerated, whereas further increases led to loss of neuronal identity, induction of senescence and apoptotic pathways, and neuronal death [31]. Finally, the largest genome-wide association study identified numerous genes participating in DNA repair which may promote the expansion of CAG repeats, including *FAN1*, *RRM2B*, *MLH1*, *MSH3*, *PMS1*, *PMS2*, and *LIG1* (Figure 1) [32].

The age at symptom onset is partly predicted by the inherited number of CAG repeats, along with other factors such as aberrant splicing and epigenetic influences (Figure 1). mRNA splicing is pivotal in HD pathogenesis [33]. Numerous studies have shown that alternative splicing, a mechanism that allows the production of multiple mRNA variants and protein isoforms from a single gene, is altered in HD [34,35,36,37]. In HD, aberrant splicing affects genes related to neuronal function, including *APPB2*, *NFASC*, and *VLDLR* [38].

On the other hand, substantial research has been developed to identify the essential epigenetic factors that contribute to the pathogenesis of HD [39]. A newly published investigation has demonstrated that hypermethylation at CpG sites in the *HTT* gene, such as cg22982173, is linked to minor progression of HD (Figure 1). Conversely, hypomethylation at some CpG sites near genes including *PEX14*, *GRIK4*, and *COX4I2*, is linked to increased motor progression in HD patients [40]. Alterations in histone acetylation (e.g., H3K9ac, H3K14ac, and H3K27ac) and histone methylation (e.g., H3K4me3, H3K9me3, and H3K27me3) indicate that mHTT protein may impact chromatin structure by promoting epigenetic alterations (Figure 1) [41].

### 1.2. Signs and Symptoms

The clinical course of HD is divided into four stages of progression [42]: (i) stage 0 (symptomless period); (ii) stage 1 (onset of neurodegeneration); (iii) stage 2 (manifestation of a recognizable clinical signs, including cognitive and/or motor manifestations of HD); (iv) stage 3 (loss of functional ability and difficulties with daily activities).

HD signs and symptoms, which generally start between the ages of 35 and 40 [43], are classified into a triad of progressive motor, cognitive, and behavioral disruptions [44]. Motor dysfunction includes involuntary movements (e.g., chorea and dystonia) and complications with voluntary movements (e.g., dysphagia and akinesia) [45]. Cognitive impairments involve a decline in executive functions, along with memory loss [46]. Neuropsychiatric problems usually include anxiety, depression, aggression, and psychosis [47]. On the other hand, motor, cognitive, and psychiatric deficits may be detected prior to the onset of HD [35]. HD typically progresses over 15–20 years following the onset of motor dysfunction, with this duration not affected by the length of CAG repeats [43].

HD has traditionally been considered to affect only the central nervous system (CNS) [30,36], but recent evidence indicates that other organs are also affected, such as the heart, bones, skeletal muscle, liver, and digestive tract [48,49,50,51]. Further issues include circadian rhythm disruptions, leading to lower sleep efficiency [52].

### 1.3. Diagnosis

HD is diagnosed through either a confirmed family history or a positive genetic test. The gold standard for this detection is the DNA analysis, which identifies a CAG repeat count in the *HTT* gene [53]. In addition to the genetic confirmation, the diagnosis requires evidence of motor disorders, as assessed by the Total Motor Score (TMS) of the Unified Huntington’s Disease Rating Scale (UHDRS). The UHDRS-TMS, with a maximum score of 124 points, includes 15 items that evaluate chorea, dystonia, parkinsonism, motor performance, oculomotor function, and balance [54].

Numerous studies are now focusing on functional changes and alterations in brain imaging before the onset of HD symptoms. Evidence suggests that changes in brain volume and neural connections can be detected several years before clinical manifestations emerge [55]. Moreover, many biomarkers (associated with oxidative stress or immune alterations) are being investigated for the early detection of HD through blood analyses [56].

### 1.4. Epidemiology

HD is found in all human populations worldwide, although its prevalence varies across regions. A relatively high prevalence can be observed in several European countries: United Kingdom (12.3 cases per 100,000) [57], France (8.9 cases per 100,000) [58], Germany (9.26 cases per 100,000) [59], and Italy (10.85 cases per 100,000) [60]. Canada also displays a high prevalence (9.33 cases per 100,000) [61]. In contrast, HD prevalence is low in Asian countries (0.65 cases per 100,000 in Japan [62] and 0.42 cases per 100,000 in China [63]). These differences in prevalence are primarily related to ethnic variation in CAG repeats and generally prevalence is higher in populations with longer average CAG repeats [64].

In addition to the HD prevalence, its socioeconomic impact profoundly increases the overall burden on the health system and influences patients and families’ quality of life [65]. This underlines the urgent need for novel and effective therapies, making research crucial for achieving better outcomes in HD patients.

Aforementioned characteristic of HD as a genetic disorder, highlights the need for a robust support system to tackle the complexity of its treatment [66]. Following section will outline most current treatment options available for HD patients.

## 2. Current Treatment Strategies

Although a cure is not currently available for HD, a range of pharmacological interventions have been suggested that provide symptom relief [67,68]. Pharmacological treatments, outlined in Table 1, should be combined with other non-pharmacological strategies to enhance disease management, such as surgical therapies and various non-invasive approaches, which collectively aim to improve functional outcomes and quality of life [69].

### 2.1. Pharmacological Therapies

This section explores the pharmacological strategies used in the management of HD, such as drugs to manage motor symptoms, psychiatric issues, and cognitive impairments.

#### 2.1.1. Dopamine-Depleting Agents

Tetrabenazine (TBZ) and deutetrabenazine (DBZ) are dopamine-depleting pharmacologic agents that inhibit the VMAT2 transporter, reducing presynaptic dopamine levels and decreasing chorea [70]. TBZ was the first agent authorized for the treatment of HD-associated chorea [71]. DBZ is an isotopic isomer of TBZ in which six hydrogen atoms in TBZ are replaced with deuterium atoms. While DBZ retains the same function as TBZ, the presence of deuterium atoms extends its half-life, allowing for less frequent administration [74].

The most typical side effects of this compound are parkinsonism, drowsiness, weakness, depression, and acute akathisia, all of which can be mitigated by reducing the dose [72]. Other side effects include insomnia, anxiety, tremors, memory issues, confusion, dizziness, and nausea [73].

#### 2.1.2. Antipsychotics

Although these drugs are primarily indicated for the treatment of psychosis, they also alleviate chorea symptoms through their interaction with the D2 receptor [75]. The following medications are utilized: (i) haloperidol (this drug selectively inhibits dopamine D2 receptors and is highly effective for treating severe chorea; clinicians continue to recommend haloperidol as the first-line antipsychotic due to its great cost-effectiveness) [76,77]; (ii) tiapride (tiapride, which blocks dopamine D2 and D3 receptors, is another cost-effective option for treating chorea in HD, with a mild and well-tolerated side effect profile) [78]; (iii) olanzapine (blocks dopamine -D1, D2, and D4-, serotonin -5-HT-; -5-HT2A and 5-HT2C-, histamine -H1-, α1-adrenergic, and muscarinic receptors; it is employed as an antipsychotic drug for managing symptoms associated with schizophrenia; recently, olanzapine has been used to treat behavioral symptoms; however, the use of this medication has led to significant side effects like dyslipidemia and weight gain) [79,80,81]; (iv) quetiapine (inhibits dopamine -D2- and 5-HT -5-HT2A- receptors; treatment with quetiapine was linked to reduction in choreiform movements and improvement in behavioral symptoms, like psychosis, agitation, irritability, and insomnia) [82,83,84]; (v) risperidone (blocks dopamine -D2- and 5-HT -5-HT2A- receptors, and is used in the management of schizophrenia; this medication improves behavioral and psychiatric assessment scores, as well as motor symptoms associated with HD; however, this drug promotes numerous side effects, like hyperprolactinemia, extrapyramidal symptoms -akathisia, parkinsonian symptoms, and acute dystonia-, weight gain, sedation, and alterations in electrical conductivity of the myocardium) [85,86,87]; (vi) apriprazole (apriprazole acts as a partial agonist at dopaminergic D2 and serotonergic 5-HT1A receptors, and as a selective antagonist at 5-HT2A receptors; it is well tolerated and has shown significant improvements in some motor and behavioral symptoms in HD patients) [88].

#### 2.1.3. Antidepressants

Antidepressants are commonly prescribed to HD patients with depression [89]. Selective 5-HT reuptake inhibitors (SSRIs) are the main antidepressants used, as they inhibit the reuptake of 5-HT into the presynaptic nerve terminal. This situation increases extracellular 5-HT levels, thus facilitating receptor interaction and producing therapeutic outcomes [77]. The following drugs are used for the treatment of HD symptoms associated with depression: (i) citalopram (the use of this medication results in an improvement in depression symptoms) [91]; (ii) escitalopram (escitalopram, like citalopram, markedly reduces depressive symptoms associated with HD) [94]; (iii) fluoxetine (alleviates depression in the short-term, but there are limited clinical trials assessing its effectiveness in HD patients, emphasizing the necessity for additional research) [95,96]; (iv) sertraline (not only alleviates depression but has also been documented to completely solve aggression and obsessive–compulsive disorder symptoms related to HD) [97].

SSRIs are associated with some side effects, such as sexual dysfunction, and weight gain [92]. Some studies have also indicated an increased risk of suicide in adolescents taking SSRIs [93]. This risk factor has not been observed in adults using SSRIs.

#### 2.1.4. Antiglutamatergics

Some studies have examined how glutamate excitotoxicity affects chorea in the context of HD [98]. Two main antiglutamatergic agents have proven to be effective: (i) amantadine (this drug is a non-competitive N-methyl-D-aspartate -NMDA- receptor antagonist; some studies have shown that amantadine provides modest benefits for HD chorea, as indicated by improvements in the UHDRS; high doses, needed for symptom relief, often lead to significant adverse effects, including hallucinations, forgetfulness, agitation, and sleepiness) [99,100,101]; (ii) riluzole (this antiglutamatergic agent has been tested for treating HD-associated chorea; it was found to reduce chorea symptoms in a dose-dependent manner, with higher doses displaying substantial effectiveness after 8 weeks of treatment; adverse effects included increased plasma levels of liver enzymes and a higher risk of suicide) [102,103].

#### 2.1.5. Anticonvulsants

To stabilize mood disorders, HD patients are prescribed anticonvulsants, including lamotrigine and carbamazepine. Multiple studies have indicated that these drugs are effective in reducing the severity of symptoms associated with HD [104,105,106]. Traditionally, anticonvulsants were used to treat seizures caused by the excessive firing of synchronized neuronal populations [107]. Anticonvulsants reduce this synchrony through three mechanisms [108]: (i) modulation of voltage-gated ion channels; (ii) upregulation of inhibitory neurotransmitters; (iii) downregulation of excitatory neurotransmitters.

The following pharmacologic agents were prescribed: (i) valproate (is an anticonvulsant that increases GABA levels in the brain; when administered in combination with olanzapine, it has been observed to help manage agitation and aggression in patients with HD; this combination allows for a reduced dosage of the antipsychotic medication, which in turn decreases the possibility of side effects) [109,110]; (ii) carbamazepine (blocks voltage-gated sodium channels -VGSCs-; although carbamazepine is prescribed as a mood stabilizer for HD, no clinical research has shown specific benefits for HD patients) [112]; (iii) lamotrigine (this medication inhibits VGSCs and blocks the release of excitatory neurotransmitters glutamate and aspartate; while lamotrigine is primarily used a mood stabilizer in HD, certain investigations indicate that its ability to reduce excitatory neurotransmitter activity and excitotoxicity, a pathological feature of HD) [115,116]; (iv) levetiracetam (binds to the synaptic vesicle protein SV2A, which disrupts the release of excitatory neurotransmitters, such as glutamate, thus blocking neuronal firing and hypersynchronization; this medication effectively reduces involuntary movements associated with HD) [117,118].

Anticonvulsants can generate numerous side effects, including hypersensitivity reactions, blood dyscrasias, gastrointestinal problems, and depression [111]. Moreover, carbamazepine may lead to severe skin conditions including Stevens-Johnson syndrome or toxic epidermal necrolysis [113,114].

### 2.2. Surgical Therapies and Non-Invasive Approaches

Surgical interventions, such as deep brain stimulation (DBS), have shown considerable promise in alleviating the symptoms of chorea in HD patients [119]. However, the invasiveness of DBS, along with the associated risks and highly specialized care required, make it a treatment option that may not be suitable for everyone [120].

A comprehensive care approach involving multiple disciplines is necessary given the wide range of symptoms associated with HD. This group of experts would include physiotherapists, occupational therapists, speech-language pathologists (SLPs), and dietitians. Physiotherapists focus on improving mobility and balance with the aim of helping to prevent falls and maintain physical function [121]. Occupational therapists collaborate with to adapt their home and work environments to satisfy their needs [122]. SLPs aim to protect both communication and swallowing capabilities [123]. Dietitians provide guidance on nutritional strategies to combat weight loss and prevent malnutrition, common features in HD [124]. Psychological sessions can be used in conjunction with pharmacological treatments to alleviate the psychological and cognitive symptoms of HD. This approach improves significantly the mental health of patients with HD [125].

Current therapies for HD are notably restricted, mainly due to their inability to alter the disease’s progression. The complex pathophysiology of HD, along with the variability in clinical manifestations and the absence of reliable biomarkers for diagnosis and monitoring, presents major challenges in developing effective treatments. Commercial treatments in HD target symptoms without affecting the cause of the disease [67,68]. Additionally, due to its gradual progression, it is difficult to observe the effects of interventions in clinical trials [126]. These limitations highlight the need for experimental therapies that target the HD etiology, including gene silencing methods which would effectively prevent neurodegenerative processes. Developing new therapies is vital for addressing the unmet medical needs of HD patients and enhancing their quality of life [67].

## 3. AAV-Delivered Genetic Targeting in Huntington’s Disease

Gene therapy (GT) is a revolutionary medical method that utilizes genetic material as a therapeutic agent to treat genetic diseases. It normally involves the introduction of functional genes to the patients’ cells or the correction of defective genes by deletion or modification of genetic codes [127]. In the GT approach, genetic materials, including DNA and RNA molecules, are introduced to the cells through delivery vehicles such as viral and non-viral vectors [128,129].

Among available viral vectors, AAVs are the most extensively employed and clinically validated platform for in vivo GT [130]. Wild-type AAVs are small, nonenveloped, and non-pathogenic viruses from the *Parvoviridae* family. Their viral genome, which is composed of a 4.7 kb single-stranded linear DNA genome, is replaced by the transgene expression cassette to generate recombinant AAVs (rAAVs) useful for GT. The expression cassette in rAAVs is flanked by two inverted terminal repeats (ITRs) and packaged in various naturally occurring AAV serotypes with specific capsid features and tropisms [131,132]. rAAVs usually remain mostly episomal in transduced cells and rarely integrate into the host genome [133]. Despite having integration reports in animals [134], the direct relationship between AAVs and genotoxicity has not been reported yet. Although rAAVs can stimulate the immune system, produce minimal serious inflammatory responses [135,136], and provide high levels of sustainable expression [137,138]. They are being used as a vector of choice in numerous clinical trials and offer successful treatments to patients suffering from different rare disorders like spinal muscular atrophy, Duchenne muscular dystrophy, and hemophilia A and B disorders [139,140,141,142,143].

AAV’s ability to transduce non-dividing cells such as neurons has made them a valuable therapeutic option for genetic CNS disorders [144,145,146]. Regardless of this, genetic material transfer to the CNS has always been a challenge [147], and administration into the cerebrospinal fluid and intraparenchymal injection are suggested to ease the burden to some extent [148,149]. Nonetheless, the problem still stands with AAV’s ability to easily cross barriers into the CNS, thus a great deal of attempts is made to improve the precision and efficacy of AAV-mediated gene delivery to different neuronal populations [150,151]. For instance, most recently, even a focused ultrasound was used to temporarily disrupt the blood–brain barrier (BBB) to improve AAV delivery into the brain of an HD mouse model [152]. Up to date, several AAV serotypes are being utilized in GT programs, with some exhibiting high brain transduction efficacy. For instance, AAV9 penetrates the BBB and efficiently transduces CNS cells [153,154]. Moreover, it has been shown that the variant AAV-PHP.B achieves successful gene transfer throughout the CNS, up to 40 times more efficiently than AAV9 [155]. Common AAV serotypes that are currently used in preclinical and clinical studies for CNS disease are discussed elsewhere [156,157].

Although rAAV-mediated GT has been widely used and investigated for treating genetic diseases in both preclinical and clinical phases, its potential for HD treatment remains largely unexplored. Generally, GT for HD treatment could be feasible by reducing the production of the toxic HTT protein in affected cells. Among various GT approaches, vector expressed editing tools have emerged as a particularly promising strategy that have been able to slow neuronal degeneration in the brain of patients and experimental animals [158]. Although promising, relatively few studies, compared with other CNS diseases, have addressed the application of rAAV-mediated GT for HD in the last two decades, highlighting a significant opportunity for further research and improvement in this area. In this section, we chronologically present significant studies, with a summary outlined in Table 2. The studies summarized in Table 2 have mainly employed various targeting strategies, including RNA interference (RNAi), short hairpin RNA (shRNA), microRNA (miRNA), zinc-finger proteins, antibodies, and CRISPR/Cas9 systems. Each approach provides unique advantages and limitations. For instance, RNAi- and miRNA-based methods usually offer robust HTT silencing but might raise concerns about off-target effects. Zinc-finger and CRISPR/Cas9 systems offer higher specificity but face challenges of editing efficiency and long-term safety. Antibody-based strategies could reduce aggregates but do not resolve transcriptional dysregulations.

At the preclinical level, it has been shown that the rAAV5 delivered GT tool for RNAi can suppress the expression of mutant HTT in the R6/1 HD transgenic mouse and ameliorate the HD phenotype [159]. Additionally, an rAAV1-delivered RNAi mechanism has also been successful in suppressing mHTT at both the mRNA and protein levels in cell culture and in HD mouse brain. This treatment was able to improve the behavioral and neuropathological abnormalities associated with HD [160].

Similarly, rAAV5-mediated delivery of RNAi into the HD model mouse striatum after the onset of disease successfully ameliorated neuropathological abnormalities by inhibiting mutant gene expression [161]. The benefits of HTT silencing were also observed in a transgenic mouse model of HD, where siRNA delivered by rAAV1/8 attenuated neuronal pathology and delayed the abnormal behavioral phenotype [162]. Furthermore, the neuroprotective efficacy of shRNA-mediated knockdown of HTT expression was shown in a study where the GT tool was delivered to rats using rAAV-HD70 [163].

The therapeutic efficacy of rAAV1 delivered nonallele-specific RNAi therapeutics for HD was reported by a significant reduction in both wild-type and mutant HTT levels in HD mice, leading to improved motor coordination and survival [164]. Supporting the use of rAAV-mediated RNAi as a therapy for HD, another study demonstrated that partial suppression of wild-type HTT expression using a rAAV2/1 delivery system was well tolerated in the non-human primate putamen, without neuronal degeneration or immune response. A 45% decrease in HTT protein levels in the mid- and caudal putamen of rhesus monkeys did not lead to motor deficits or neuronal degeneration [165]. In HD patient iPSC-derived neuronal cultures, the efficacy of rAAV5-miHTT was demonstrated by a reduction in HTT mRNA and protein levels, without off-target effects in gene expression and regulation in neuronal cells and astrocytes [166].

Furthermore, the potential of rAAV2/1 as a delivery tool for synthetic zinc finger that suppresses mHTT expression at both mRNA and protein levels in the brain of the R6/2 HD mouse model was also addressed [167]. Bilateral delivery of an rAAV2 vector encoding an anti-HTT short hairpin RNA to the striatum of rhesus monkeys resulted in a reduction in HTT mRNA and protein levels without side effects up to 6 months post administration [168]. Intrajugular vein administration of rAAV9 expressing a mutant HTT-specific RNAi construct was proven effective in reducing mHTT expression in multiple brain regions and peripheral tissues affected in mice [169].

Another study reported the benefits of rAAV2/1-mediated RNAi in reducing both wild-type and mutant HTT in striatum cells of the YAC128 mouse model of HD. This treatment also led to improvements in behavioral deficits, and a reduction in HTT aggregation, without significant neurotoxicity following intracranial injections [170]. Similar findings were reported in several HD mouse models following one-time striatal rAAV-ZFP application, which led to HTT-lowering and improvement in histopathological, electrophysiological and biomarker deficits [171,172]. Another group of scientists highlighted the high potential of rAAV1 and rAAV2 to transduce the cortico-striatal tissues predominantly affected in HD in the non-human primate brain, suggesting RNAi-based therapy for targeting neurons that degenerate in HD [173].

The design of artificial microRNA-expression constructs, their incorporation into rAAV5, their total silencing of both wild-type and mtHTT, and their allele-specific silencing have been investigated and reported in vitro and in the humanized transgenic Hu128/21 HD mouse model [174]. Moreover, AAVs have also been found beneficial in developing antibody-based therapies for HD. For instance, rAAV6-INT41 was shown to reduce mHTT in the R6/2 mouse model [175,176]. The practicability of total and allele-selective HTT silencing induced by therapeutic microRNAs delivered in rAAVs to the brain without safety concerns has also been demonstrated. Researchers reported the successful suppression of mutant HTT aggregate formation in HD rats by intracerebral administration of rAAV5-miHTT-155. While this construct showed high efficiency, rAAV5-miSNP50T was proven to be more precise in mutant HTT allele selectivity [177].

Meanwhile, the rAAV-delivered CRISPR/Cas9 based edition of the mutant HTT allele was also reported to reduce human mutant expression in the treated hemispheres of BacHD mice [178]. Furthermore, it was shown that an rAAV-delivered CRISPR/Cas9-mediated system can suppress endogenous mHTT expression in the striatum of mHTT-expressing mice and mitigate neuropathology in the adult brain [179]. The therapeutic value of a single intracranial administration of rAAV5-miHTT for decreasing HTT levels of mRNA and protein was validated in a large animal model, a transgenic HD (tgHD) minipig model, in which both mRNA and protein levels of HTT were reduced in the transduced regions of the brain [180].

Disruption in the expression of the m*HTT* gene in the striatum of the R6/2 mouse model of HD was reported using the rAAV-delivered CRISPR-Cas9 system, resulting in decreased neuronal inclusions and improvements in lifespan and certain motor deficits [181]. It is worth mentioning that rAAV5-miHTT can reduce HTT protein in the striatum and cortex of Q175 HD mice in a dose-dependent manner, emphasizing the benefits of rAAV-mediated HTT-lowering GT for HD [182]. Non-targeted HTT downregulation with rAAV5-miHTT in the humanized Hu128/21 mouse model of HD was sustained for seven months, as addressed by Caron et al. (2020) [183].

A significant reduction in HTT gene expression in deeper tissues and outer layers of the brain of non-human primates by a gene therapy candidate, VY-HTT01, encapsulated in an rAAV1 delivering RNAi mechanism was presented at the 2018 edition of the ESGCT congress [184,185]. The safety and tolerability of rAAV5-miHTT in the non-human primate *Macaca fascicularis* and Sprague-Dawley rats were demonstrated by intrastriatal administration, with widespread vector DNA and miHTT transgene distribution in the brain areas associated with HD pathology [186]. The widespread biodistribution and durable efficiency of rAAV5-miHTT were also shown in HD-relevant regions of the minipigs brain [187]. There is also a report on the application of rAAV1 and rAAV2 in vitro and in vivo for HTT gene targeting using primary artificial miRNA (pri-amiRNA) delivered to numerous species, such as mice and non-human primates [188]. rAAV5-miHTT-mediated HTT suppression was reported to benefit brain health in an HD mouse model [189]. The neuroprotective effect of hepatoma-derived growth factor (HDGF) in HD treatment was described by employing rAAV8-mediated delivery of HDGF to the striatum of an HD mouse model, which could reduce mHTT; however, there were no significant changes in neurological phenotypes [190]. Finally, most recently, rAAV5 delivered engineered microRNA targeting the HTT exon 1 sequence (rAAV5-miHTT), could significantly reduce HTT mRNA and protein levels in the brain of HD mouse models, heterozygous zQ175 knock-in mice and humanized Hu128/21 mice [191]. Additional studies reporting the benefits of various rAAV serotypes to the HD treatment, along with insights into the disease’s mechanisms and pathogenesis are outlined elsewhere [194,195,196,197,198,199,200,201,202,203,204,205,206,207,208,209,210,211].

At the clinical level, the Phase I/II clinical study (clinical trial No. NCT04120493), a total HTT-lowering therapy, investigates the safety, tolerability, and efficacy of rAAV5-miHTT in adults with early manifest HD [192], which is widely described elsewhere [212]. The safety and efficacy of AMT-130 in European adults is addressed in the clinical trial No. NCT05243017 [213]. Additionally, the phase I/II clinical trial No. NCT05541627, evaluates the safety, tolerability, and preliminary efficacy of one-time intracerebral bilateral injections of AB-1001 (AAVrh10.CAG.hCYP46A1) in adults with early manifest HD. This rAAV-mediated GT aims to express the human cholesterol 24-hydroxylase gene within the striatum of individuals [193].

The GT studies mentioned here use different mechanisms to lower mutant HTT or reduce its toxic effects. Several approaches, including those using RNAi, shRNA, and miRNA, silence HTT mRNA, leading to lower protein levels and fewer aggregates. Zinc-finger proteins and CRISPR/Cas9 act directly at the DNA level, and this offers higher specificity but raises long-term safety questions. Antibody-based methods aim to neutralize toxic protein fragments rather than preventing their production. Altogether, these approaches reduce mutant HTT burden and improve motor, cognitive, or survival outcomes in models, showing both promise and the challenges that remain for translation to patients.

## 4. Future Directions

AAVs have shown significant promise in the treatment of genetic and rare diseases, successfully bringing several therapeutic options from the lab to the patient’s bedside. Currently, many preclinical and clinical trials are being conducted for the treatment of genetic diseases by AAVs, with neurodegenerative diseases attracting growing interest for their therapeutic possibilities. Among therapeutic options, AAVs ability to transduce various CNS cells has made them valuable therapeutic and research tools for CNS disorders such as HD. AAV-delivered therapeutic transgenes remain episomal in target cells, which ensures their stability in non-dividing cells and long-term expression of therapeutic tools. This characteristic is particularly advantageous in the treatment of HD, in which the primary concern is with non-dividing cells, specifically neurons. Consequently, a single dose administration may provide a lifelong therapy for HD patients [158]. It is worth mentioning that recent clinical trials with antisense oligonucleotides (ASOs), such as those targeting HTT mRNA, have not shown sustained efficacy in HD, mainly due to limited CNS penetration and the need for repeated dosing [214]. In contrast, AAV-based GT may overcome these obstacles by enabling widespread CNS transduction and long-term expression of therapeutic material after a single administration.

As noted in the literature review, AAVs are not only useful in HD modeling [163,215,216,217], but they also hold a great deal of promise for treating HD. Generally, AAV-mediated therapeutic strategies for treating HD generally focus on the goal of reducing toxic HTT protein production. Reducing mutant HTT protein alleviates toxic effects, but its complete elimination is not recommended due to the critical functions of wild-type HTT, which include its key role in transcription regulation, normal mitochondrial activity, and protecting brain cells from apoptosis. Furthermore, its absence may lead to embryonic lethality and neurodegeneration. Meanwhile, the long-term consequences of HTT reduction in various organs of the human body, including the CNS, remain poorly understood [9,158,218,219,220,221]. In general, HTT reduction appears to have a safe and favorable profile, particularly when partially reduced [222].

On the other hand, despite its potential, particular challenges are also present in the developmnt of AAV-mediated HD treatment, originating from HD characteristics themselves and AAVs’ limitations. Efficient transduction of CNS cells as previously mentioned, correct tissue targeting, vector production challenges and limited cloning capacity of AAV (approximately 4.5–4.7 kb), immune response and safety precautions are among the important challenges. Advantages and disadvantages of common routes of AAV delivery to the CNS, including intraparenchymal delivery, intravenous delivery, and delivery to cerebrospinal fluid, are widely outlined elsewhere [149]. Moreover, the immune system’s response to AAV capsids and transgene products is well discussed before [136]. Another significant barrier in HD treatment by AAVs is the absence of an adequate animal model that accurately mimics the human condition by expressing both normal and mutant HTT at physiological levels [158]. It seems that a combination of comprehensive in vitro and in vivo studies is still needed to shed light on the practical aspects of HD treatment using AAVs as a GT delivery system. The translation of current knowledge into meaningful clinical improvements in affected patients is still lacking. The authors hope that the current review will help further unlock the full potential of AAVs in treating HD.

## Figures and Tables

**Table 1:** List of commonly used drugs for treating symptoms associated with HD.

Drug Category	Compound	Mechanisms of Action	References
Dopamine-depleting agents	Tetrabenazine (TBZ)	Blockade of VMAT2 transporter	[70,71,72,73]
	Deutetrabenazine (DBZ)		[70,72,73,74]
Antipsychotics	Haloperidol	Blockade of D2 receptor	[75,76,77]
	Triapride	Blockade of D2 and D3 receptors	[75,78]
	Olanzapine	Blockade of D1, D2, and D4 receptors Blockade of 5-HT2A and 5-HT2C receptors Blockade of H1 receptor Blockade of α1-adrenergic receptor Blockade of muscarinic receptors	[75,79,80,81]
	Quetiapine	Blockade of D2 receptor Blockade of 5-HT2A receptor	[75,82,83,84]
	Risperidone	Blockade of D2 receptor Blockade of 5-HT2A receptor	[75,85,86,87]
	Apriprazole	Partial agonist at D2 receptor Partial agonist at 5-HT1A receptor Blockade of 5-HT2A receptor	[75,88]
Antidepressants (SSRIs)	Citalopram	Inhibition of 5-HT reuptake	[89,90,91,92,93]
	Escitalopram		[89,90,92,93,94]
	Fluoxetine		[89,90,92,93,95,96]
	Sertraline		[89,90,92,93,97]
Antiglutamatergics	Amantadine	Blockade of NMDA receptor	[98,99,100,101]
	Riluzole		[98,102,103]
Anticonvulsants	Valproate	Elevation of GABA levels in the synaptic cleft	[104,105,106,107,108,109,110,111]
	Carbamazepine	Blockade of VGSCs	[104,105,106,107,108,111,112,113,114]
	Lamotrigine	Blockade of VGSCs Inhibition of the release of glutamate and aspartate from storage	[104,105,106,107,108,111,115,116]
	Levetiracetam	Interaction with SV2A protein Inhibition of neurotransmitter release from storage	[104,105,106,107,108,111,117,118]

**Table 2:** Chronological overview of rAAV-mediated GT studies for HD.

AAV Serotype	Therapy Mechanism	Species/Model	Outcome	References
AAV5	RNAi	R6/1 HD mouse	Suppressed mutant HTT Ameliorated HD phenotype	[159]
AAV1		HD mouse	Improved behavioral and neuropathological abnormalities	[160]
AAV5		HD mouse	Ameliorated neuropathological abnormalities	[161]
AAV1/8	siRNA	Transgenic HD mouse	Attenuated neuronal pathology Delayed abnormal behavioral phenotype	[162]
AAV-HD70	shRNA	Rat	Neuroprotective efficacy demonstrated	[163]
AAV1	RNAi	HD mouse	Improved motor coordination and survival	[164]
AAV2/1	RNAi	Rhesus monkey	45% reduction in HTT protein No motor deficits or neuronal degeneration	[165]
AAV5	miRNA	Human iPSC	Reduction in HTT mRNA and protein levels No off-target effects	[166]
AAV2/1	Zinc-finger protein	R6/2 HD mouse	Suppressed mutant HTT expression	[167]
AAV2	shRNA	Rhesus monkey	Reduction in HTT mRNA and protein levels No side effects up to 6 months	[168]
AAV9	RNAi	Mouse	Reduced mHTT expression in multiple brain regions and peripheral tissues	[169]
AAV2/1	RNAi	YAC128 mouse	Reduced HTT aggregation Improved behavioral deficits No significant neurotoxicity	[170]
AAV2/1 AAV6	Zinc-finger protein	HD mouse models	HTT-lowering Improved histopathological, electrophysiological, and biomarker deficits	[171,172]
AAV1 AAV2	RNAi	Non-human primate	High transduction of cortico-striatal tissues	[173]
AAV5	miRNA	Hu128/21 HD mouse	Total and allele-specific silencing of HTT	[174]
AAV6	Antibody (INT41)	R6/2 mouse	Reduced mHTT	[175,176]
AAV5	miRNA	HD rat models	Suppressed mutant HTT aggregate formation	[177]
AAV2/1	CRISPR/Cas9	BacHD mouse	Reduced human mutant HTT expression	[178]
rAAV	CRISPR/Cas9	mHTT-expressing mouse	Suppressed mHTT expression Attenuated neuropathology	[179]
AAV5	miRNA	tgHD minipig	Reduced HTT mRNA and protein levels	[180]
AAV1	CRISPR-Cas9	R6/2 mouse	Decreased neuronal inclusions Improved lifespan and motor deficits	[181]
AAV5	miRNA	Q175 HD mouse	Dose-dependent reduction in HTT protein in striatum and cortex	[182]
AAV5	miRNA	Hu128/21 mouse	Sustained non-selective HTT reduction for 7 months	[183]
AAV1	RNAi	Non-human primates	Significant reduction in HTT gene expression in brain tissues	[184,185]
AAV5	miRNA	*Macaca fascicularis*, Sprague-Dawley rat	Safety and tolerability demonstrated Widespread vector distribution	[186]
AAV5	miRNA	Minipig	Widespread biodistribution and durable efficiency in HD-relevant brain regions	[187]
AAV1 AAV2	pri-amiRNA	Mouse Non-human primates	HTT targeting in various species	[188]
AAV5	miRNA	HD mouse model	HTT suppression benefited brain health	[189]
AAV8	HDGF	HD mouse model	Reduced mHTT No significant changes in neurological phenotypes	[190]
AAV5	miRNA	zQ175 knock-in mouse Hu128/21 mouse	Significant reduction in HTT mRNA and protein levels	[191]
AAV5	miRNA	Human	Safety, tolerability, and efficacy explored in adults with early manifest HD	[192]
AAVrh10	Gene expression	Human	Safety, tolerability, and preliminary efficacy in adults with early manifest HD	[193]

## Data Availability

Not applicable. No new data were generated.

## Abbreviations

The following abbreviations are used in this manuscript:

5-HT	Serotonin
5mC	5-methylcytosine
AAV	Adeno-associated virus
APPB2	Amyloid beta precursor protein binding protein 2
ASO	Antisense oligonucleotide
BBB	Blood–brain barrier
CBP	CREB-binding protein
CNS	Central nervous system
COX4I2	Cytochrome c oxidase subunit 4I2
CRISPR/Cas9	Clustered regularly interspaced short palindromic repeats/CRISPR-associated protein 9
DBS	Deep brain stimulation
DBZ	Deutetrabenazine
ESGCT	European Society of Gene and Cell Therapy Annual Congress
FAN1	FANCD2/FANCI-associated nuclease 1
GABA	γ-aminobutyric acid
GRIK4	Glutamate ionotropic receptor kainate type subunit 4
GT	Gene therapy
HC	Huntington’s chorea
HD	Huntington’s disease
HDGF	Hepatoma binding growth factor
HEAT	Huntingtin, elongation factor 3, protein phosphatase 2A, and the yeast kinase TOR1
HTT	Huntingtin
HTTNT	Huntingtin nuclear transport
iPSC	Induced pluripotent stem cell
ITR	Inverted terminal repeat
LIG1	DNA ligase 1
mHTT	Mutated huntingtin protein
miHTT	MicroRNA targeting huntingtin
miRNA	MicroRNA
MLH1	MutL homolog 1
MSH3	MutS Homolog 3
NFASC:	Neurofascin
NMDA	N-methyl-D-aspartate
PEX14	Peroxisomal biogenesis factor 14
PMS1	PMS1 protein homolog 1
PMS2	PMS1 protein homolog 2
polyQ	Polyglutamine
PRD	Proline-rich sequence recognition domains
pri-amiRNA	Primary artificial microRNA
RNAi	RNA interference
RRM2B	Ribonucleotide reductase regulatory TP53 inducible subunit M2B
shRNA	Short hairpin RNA
siRNA	Small interfering RNA
SLP	Speech-language pathologist
SSRI	Selective serotonin reuptake inhibitor
SV2A	Synaptic vesicle glycoprotein 2A
TBZ	Tetrabenazine
TMS	Total motor score
UHDRS	Unified Huntington’s Disease Rating Scale
VGSC	Voltage-gated sodium channel
VLDLR	Very low-density lipoprotein receptor
VMAT2	Vesicular monoamine transporter 2
ZFP	Zinc finger protein
